# Differential remodelling of mitochondrial subpopulations and mitochondrial dysfunction are a feature of early stage diabetes

**DOI:** 10.1038/s41598-022-04929-1

**Published:** 2022-01-19

**Authors:** Bodour S. Rajab, Sarah Kassab, Connor D. Stonall, Hussam Daghistani, Stephen Gibbons, Mamas Mamas, David Smith, Aleksandr Mironov, Zainab AlBalawi, Yin Hua Zhang, Florence Baudoin, Min Zi, Sukhpal Prehar, Elizabeth J. Cartwright, Ashraf Kitmitto

**Affiliations:** 1 Division of Cardiovascular Sciences, School of Medical Sciences, Faculty of Biology, Medicine and Health, The University of Manchester, Manchester Academic Health Science Centre, AV Hill, Dover Street, Manchester, M13 9PL UK; 2 Faculty of Applied Medical Sciences, Umm Al-Qura University, Mecca, Saudi Arabia; 3 King Abdulaziz University, Jeddah, Saudi Arabia; 4 Keele Cardiovascular Research Group, Centre for Prognosis Research, Institute of Primary Care and Health Sciences, Keele University, Stoke-on-Trent, UK; 5 EM Core Facility (RRID: SCR_021147), Faculty of Biology, Medicine and Health, The University of Manchester, Manchester, UK; 6 Seoul National University, College of Medicine, Seoul, Korea; 7 Yanbian University Hospital, Jilin, China

**Keywords:** Cardiovascular biology, Metabolism, Electron microscopy, Biochemistry, Cardiovascular biology

## Abstract

Mitochondrial dysfunction is a feature of type I and type II diabetes, but there is a lack of consistency between reports and links to disease development. We aimed to investigate if mitochondrial structure–function remodelling occurs in the early stages of diabetes by employing a mouse model (GENA348) of Maturity Onset Diabetes in the Young, exhibiting hyperglycemia, but not hyperinsulinemia, with mild left ventricular dysfunction. Employing 3-D electron microscopy (SBF-SEM) we determined that compared to wild-type, WT, the GENA348 subsarcolemma mitochondria (SSM) are ~ 2-fold larger, consistent with up-regulation of fusion proteins Mfn1, Mfn2 and Opa1. Further, in comparison, GENA348 mitochondria are more irregular in shape, have more tubular projections with SSM projections being longer and wider. Mitochondrial density is also increased in the GENA348 myocardium consistent with up-regulation of PGC1-α and stalled mitophagy (down-regulation of PINK1, Parkin and Miro1). GENA348 mitochondria have more irregular cristae arrangements but cristae dimensions and density are similar to WT. GENA348 Complex activity (I, II, IV, V) activity is decreased but the OCR is increased, potentially linked to a shift towards fatty acid oxidation due to impaired glycolysis. These novel data reveal that dysregulated mitochondrial morphology, dynamics and function develop in the early stages of diabetes.

## Introduction

Cardiovascular disease is a leading cause of mortality in both type I and type II diabetes^1^. There is a large body of evidence to indicate that a hallmark of both types of diabetes is the development of cardiac mitochondrial dysfunction^2^, ranging from impaired oxidative phosphorylation (OXPHOS), substrate inflexibility, aberrant mitochondrial dynamics and mitophagy; mediated by an array of complex, interlinked signalling pathways that have yet to be fully delineated (for a recent review see Gollmer et al.^3^). Importantly, adverse mitochondrial remodelling is suggested to play a central role in the development of impaired cardiac function in diabetic patients^3^.

There are numerous reports describing changes to mitochondrial morphology in models of cardiovascular disease and patients (in both the presence and absence of diabetes) with contrasting evidence for mitochondrial swelling and fragmentation^4^. Mitochondrial size and distribution are mediated by proteins regulating mitochondrial dynamics, namely fission and fusion, mitochondrial motility and clearance (mitophagy); processes essential for maintaining mitochondrial quality control^5^. Fusion, the merging of two mitochondrion, occurs in two-steps; fusion of the outer membrane (OMM) driven by mitofusin 1 (Mfn1), mitofusin 2 (Mfn2), followed by fusion of the inner membrane (IMM) regulated by optic atrophy gene, Opa1. Fission of a single mitochondrion into two smaller mitochondria occurs through the recruitment of the cytosolic protein Dynamin-related protein 1, (Drp1) to the OMM. Mitophagy, the removal of damaged mitochondria, is linked to the balance between fission and fusion mechanisms and is mediated by PTEN-induced putative kinase protein 1 (PINK1)^6,7^ which recruits Parkin to the OMM^8,9^. Parkin is phosphorylated by PINK1^8,10^ culminating in the ubiquitination and subsequent degradation of OMM proteins. As reviewed by Forte et al.^5^ there are multiple reports of expression level changes to proteins mediating mitochondrial dynamics across a range of cardiovascular disorders with strategies to promote fusion, or inhibit fission, having suggested therapeutic applications.

Unlike non-muscle cells, cardiac mitochondria can be grouped into spatially distinct populations; subsarcolemmal mitochondria (SSM), interfibrillar mitochondria (IFM) and perinuclear mitochondria (PNM). Studies suggest that these populations are functionally heterogeneous for example, IFM are associated with increased respiration rates for driving neighbouring sarcomeric contraction^11,12^. There are also reports of mitochondrial subpopulations being differentially affected by pathological stress with the SSM linked to higher levels of ROS generation^13^. 3-D electron microscopy methods have revealed that the SSM, IFM and PNM form a complex inter-connected network throughout the cardiomyocyte; although the formation of a mitochondrial continuum does not necessarily negate spatially organised mitochondria having differing roles or varying stress responses^14^.

The majority of studies have investigated mitochondrial dysfunction in animal models and diabetic patients exhibiting both hyperglycemia and hyperinsulinemia. Here, to investigate changes to mitochondrial function and morphology in the early stages of diabetes and the effects of hyperglycemia upon mitochondrial morphology and dynamics, we employed the GENA348 mouse, a model of Maturity Onset Diabetes in the Young (MODY), first identified in patients in the 1970s, with a genetic basis^15^. A common MODY mutation occurs in the glucokinase (*Gck*) gene^16^. The GENA348 mouse recapitulates a *Gck* I366F mutation leading to impaired enzymatic activity rendering it unable to phosphorylate glucose to glucose-6-phosphate^17^, an essential step in the glycolysis pathway. MODY patients typically exhibit hyperglycaemia but are not insulin resistant, and thus the GENA348 mouse represents a novel model to investigate early changes to mitochondrial function and importantly, new insights into the early progression of other more pathogenic forms of diabetes.

We have employed the GENA348 mouse at 6 months of age, exhibiting signs of early left ventricular dysfunction indicative of mild heart failure. Combining biochemical and imaging techniques, serial block face scanning electron microscopy (SBF-SEM)^18,19^ and transmission electron microscopy (TEM), we have determined that the GENA348 mouse heart exhibits mitochondrial structural and functional remodelling with an imbalance between mitochondrial fission and fusion, and evidence of stalled mitophagy.

## Results

### GENA348 mice are hyperglycemic but have normal insulin levels

Transthoracic echocardiography revealed that the GENA348 animals (fed on the same diet and housed under the same conditions as aged-matched (6 months old) wild-type (WT) mice), exhibit increased left ventricular (LV) dimensions (dPW and sPW) and increased LV mass and right wall thickness. A reduction in early-to-late (E:A) ventricular filling ratio and an increase in IVRT was also observed (Table 1). These data indicate the GENA348 mice have developed cardiac hypertrophy and mild LV dysfunction. The heart weight:tibia length is increased in the GENA348 mice which is also indicative of cardiac hypertrophy. Further, in keeping with general observations of *Gck-*MODY2 patients, blood glucose levels were elevated but with no change to serum insulin (Table 1). As also reported in Table 1 there is no change to lung weight which indicates that pulmonary congestion is not a feature of the GENA348 mouse, consistent with this model being one of ‘mild’ diabetes with early stage cardiac dysfunction^20^.

**Table 1:** Summary of echocardiography and physiological parameters in GENA348 mice compared to WT (6 months of age).

Echocardiography	WT n = 15	GENA348 n = 8	*P* value
dLVD (mm)	4.57 ± 0.06	4.43 ± 0.09	0.24
sLVD (mm)	3.47 ± 0.08	3.27 ± 0.09	0.15
dIVS (mm)	0.98 ± 0.03	1.03 ± 0.03	0.37
sIVS (mm)	1.41 ± 0.03	1.42 ± 0.06	0.82
dPW (mm)	1.06 ± 0.03	1.28 ± 0.06	0.002
sPW (mm)	1.31 ± 0.03	1.54 ± 0.07	0.0006
HR (b/min)	469 ± 8.0	456 ± 4.0	0.34
LV mass/(mg)	194 ± 7.0	222 ± 11	0.05
RWT	0.45 ± 0.01	0.53 ± 0.02	0.02
EF%	55 ± 1.90	59.5 ± 2.1	0.22
FS%	24.2 ± 1.2	26. 3 ± 1.3	0.28
E:A	2.65 ± 0.20	1.84 ± 0.19	0.03
IVRT	15.2 ± 1.13	20.5 ± 0.37	0.004

Physiological	WT	GENA348	*P* value
Blood glucose (fasted) (mmol/l)	7.56 ± 0.39 (n = 5)	9.583 ± 0.38 (n = 6)	0.005
Insulin (µg/ml)	2.26 ± 0.12 (n = 5)	2.07 ± 0.10 (n = 6)	0.09
HW/TL (mg/mm)	6.56 ± 0.17 (n = 15)	7.37 ± 0.17 (n = 13)	0.0022
LW (mg)	161.10 ± 4.54 (n = 15)	169.20 ± 4.60 (n = 13)	0.2233

*dLVD* diastolic diameter, *sLVD* systolic diameter, *dIVS* diastolic intraventricular septum, *sIVS* systolic intraventricular septum, *dPW* diastolic posterior wall, *sPW* systolic posterior wall, *HR* heart rate, *EF* ejection fraction, *FS* fractional shortening, *LV mass* left ventricular mass, *RWT* relative wall thickness, *E:A* early to late ventricular filling ratio, *IVRT* isovolumetric relaxation time, *HW* heart weight, *TL* tibia length, *LW* lung weight. Data are presented as the mean ± SEM (*P* < 0.05 considered as a significant change).

### GENA348 cardiac mitochondria exhibit morphological changes compared to WT

**Figure 1:**
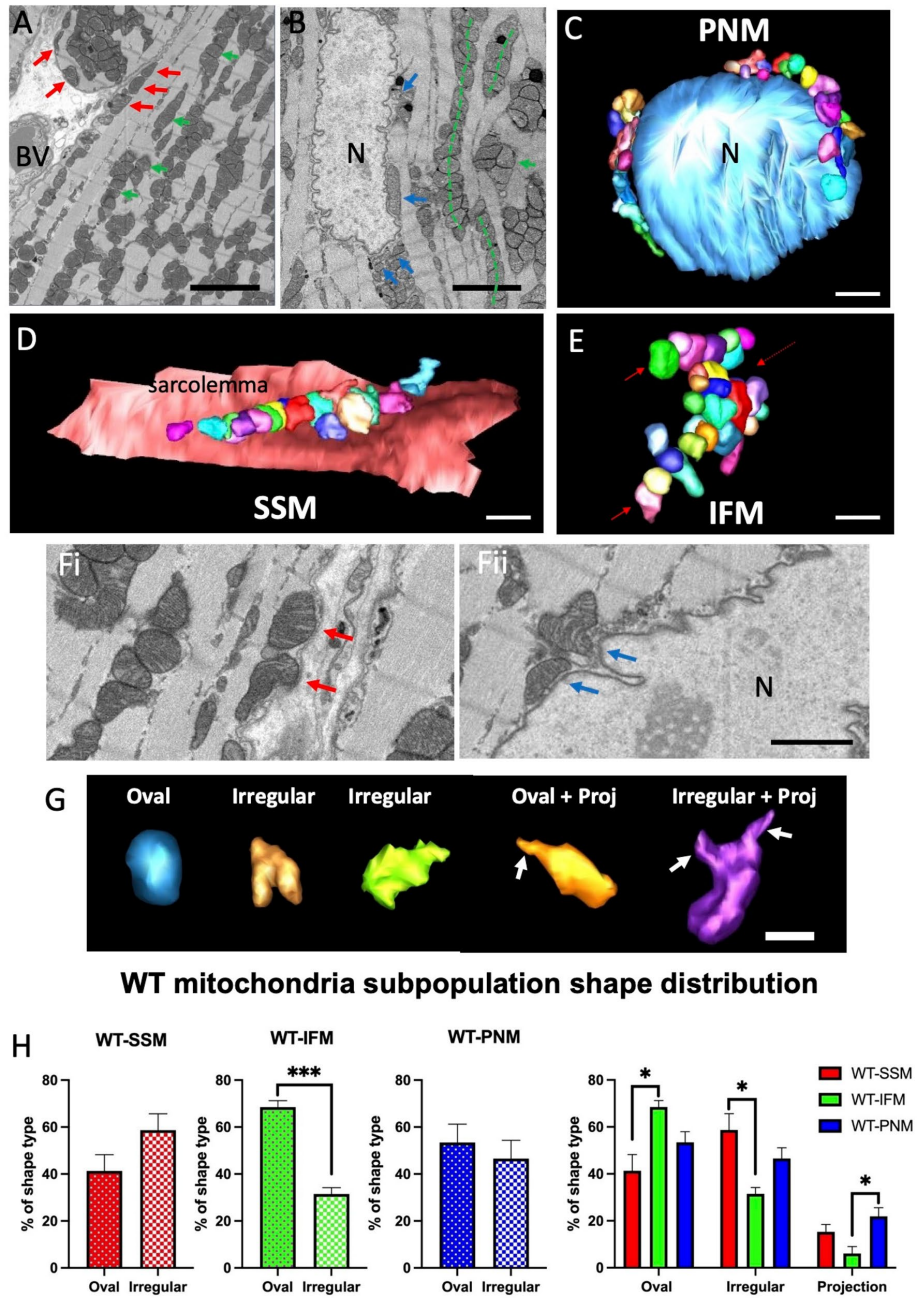
Spatial organisation of mitochondria in the myocardium (apex) of wild-type (WT) mice. (**A**) Portion of a serial image taken from an SBF-SEM data stack; red arrows indicate SSM, green arrows indicate IFM. *BV* blood vessel. (**B**) Area of a serial image showing a cardiomyocyte nucleus (N) with blue arrows indicating PNM. The dashed green lines indicate how IFM form rows of mitochondria. Scale bar = 5 µm. (**C**) 3-D Segmentation of part of a nucleus (light blue isosurface) and PNMs at the poles of the nucleus either directly in contact or juxtaposed with the nuclear envelope (individual mitochondria are displayed in a range of colours). (**D**) A cluster of segmented SSM directly adjacent or touching the sarcolemma (pink isosurface). (**E**) Examples of segmented IFMs showing that rows are also interconnected. Scale bar = 2 µm. (**Fi**,**ii**) Indicates how SSM and PNM follow the topography of the adjacent membrane which may explain why their shapes are generally more irregular than PNM. Scale bar = 2 µm. (**G**) Examples of WT 3-D reconstructed mitochondria and different shapes. Arrows indicate projections. Scale bar = 1 µm. (**H**) Analysis of the proportion of each shape type (oval, irregular and mitochondria with a tubular projection) within each WT subpopulation. Scale bar = 1 µm.

We used SBF-SEM to investigate the morphology of the mitochondria within the WT and GENA348 myocardium. Segmented mitochondria were classified according to their spatial location within the cardiomyocyte as shown in Fig. 1A,B. SSM were defined as located immediately under the sarcolemma, IFM as those flanked on either side by myofibrils, and PNM directly adjacent to the nuclear envelope. SSM and PNM that merged with IFM were not included; see Fig. 1C–E for examples of 3-D reconstructed WT mitochondria. In general the shapes of the WT IFM which form ‘strands’ appeared more oval, while WT SSM and WT PNM often matched the sarcolemma topography which may account for irregularly shaped mitochondria (Fig. 1F); although the undulating membrane topology may be influenced by the irregular mitochondrial shape. To quantify this observation, we classified each segmented (3-D reconstructed) mitochondrion into 3-shape categories; oval, irregular and mitochondria with tubular projections (including oval and irregularly shaped mitochondria); Fig. 1G. We determined that there are (i) more than twice as many oval WT IFM compared to irregular shapes (69 ± 3%, oval; 31 ± 3%, irregular; *P* = 0.0006). (ii) WT IFM have a greater proportion of oval mitochondria compared to SSM (*P* = 0.0109) and (iii) WT PNM are more likely to have a tubular projection compared to IFM (*P* = 0.0329) (Fig. 1H).

**Figure 2:**
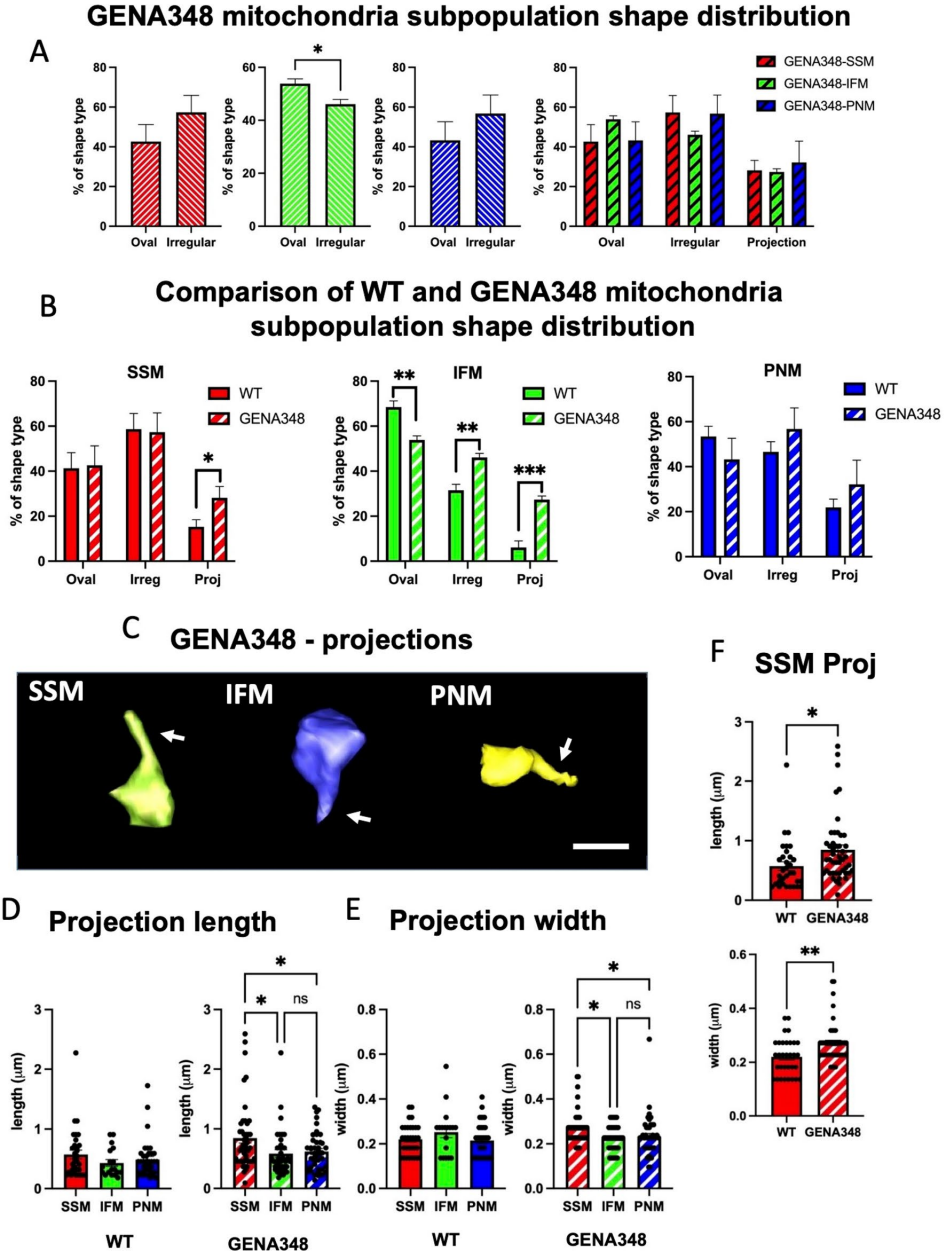
A comparison of the WT SSM, IFM and PNM shape types within GENA348 mitochondrial subpopulations. (**A**) Distribution of the shape type within the GENA348 mitochondrial population. (**B**) A comparison of the WT and GENA348 shape types reveals that GENA348 SSM and IFM have more mitochondria with tubular projections compared to the WT and that there are more irregularly shaped IFM. (**C**) Examples of 3D reconstructions of GENA348 mitochondria with tubular projections. Scale bar = 1 µm. (**D**) Tubular projections from WT mitochondria are the same length independent of mitochondrial subtype whereas GENA348 SSM have longer projections compared to IFM and PNM. (**E**) The width of the WT tubular projections are the same across each mitochondrial group whereas in the GENA348 SSM are wider than the IFM and PNM (raw data points show to spread of measurements, n = 3). (**F**) A comparison of the projection parameters (raw data points shown, n = 3) identified the GENA348 SSM as being longer and wider than WT SSM. There was no difference in IFM and PNM dimensions between the WT and GENA348 (not shown). Projection dimensions are reported in Table 2.

We next segmented similar numbers of SSM, IFM and PNM from the GENA348 SBF-SEM datasets (~ 800 mitochondria; n = 3) using the same criteria as for WT. Shape classification of the subpopulations revealed there are similar proportions of oval:irregular GENA348 SSM and PNM as within the WT subpopulations (Fig. 2A,B) and more oval types in the IFM population. However, while the majority of GENA348 IFM are oval (*P* = 0.0364) the mean ratio of oval to irregular is 1.2 (Fig. 2B), compared with 2.2 in WT i.e. there are relatively less oval GENA348 IFM (Fig. 1H). Moreover, the GENA348 subpopulations have a similar percentage of mitochondria with tubular projections, SSM, 28 ± 5%; IFM, 27 ± 2%; PNM 32 ± 11%; in contrast WT IFM are less likely to have projections than WT PNM. Significantly, the GENA348-IFM have more projections compared to WT-IFM (*P* = 0.0475), as do the GENA348 SSM (*P* < 0.0001), Fig. 2B. Examples of GENA348 mitochondria with tubular projections are shown in Fig. 2C. As presented in Fig. 2D,E and Table 2 the lengths and widths (measured close to the furthermost tip) of the projections in the WT subpopulation are similar, whereas within the GENA348 subpopulations the SSM tubular extensions are longer than IFM and PNM. Further, the GENA348 SSM projections are longer and wider than those of WT-SSM (Fig. 2F); there was no difference between WT and GENA348 IFM and PNM subgroups (not shown). These data indicate that in this model of early stage diabetes (i) there are more irregularly shaped IFM, (ii) projections are a feature of all WT and GENA348 mitochondria subtypes but that (iii) the GENA348 SSM have more tubular projections than WT SSM and are longer and wider.

**Table 2:** Summary of mitochondrial morphological features.

	SSM	IFM	PNM
**Dimensions of mitochondrial tubular projections**			
Wild type (WT)			
No. of projections	32	20	39
Length (µm)	0.574 ± 0.415	0.432 ± 0.242	0.488 ± 0.316
Width (µm)	0.220 ± 0.068	0.253 ± 0.106	0.215 ± 0.075
GENA348			
No. of projections	48	39	46
Length (µm)	0.847 ± 0.556	0.585 ± 0.391	0.618 ± 0.322
Width (µm)	0.271 ± 0.073	0.225 ± 0.057	0.232 ± 0.014
**Cristae parameters**			
Wild type (WT)			
No. of cristae	278	301	195
W_O_ (nm)	34 ± 3	36 ± 3	38 ± 6
W_I_ (nm)	16 ± 1	16 ± 1	17 ± 3
S (nm)	28 ± 3	27 ± 4	29 ± 3
GENA348			
No. of cristae	302	310	255
W_O_ (nm)	34 ± 6	37 ± 3	38 ± 1
W_I_ (nm)	16 ± 2	17 ± 2	17 ± 1
S (nm)	23 ± 5	25 ± 2	26 ± 2

N = 3 per group. Data are presented as the mean ± s.d.

### PNM nanotunnels in the GENA348 myocardium

**Figure 3:**
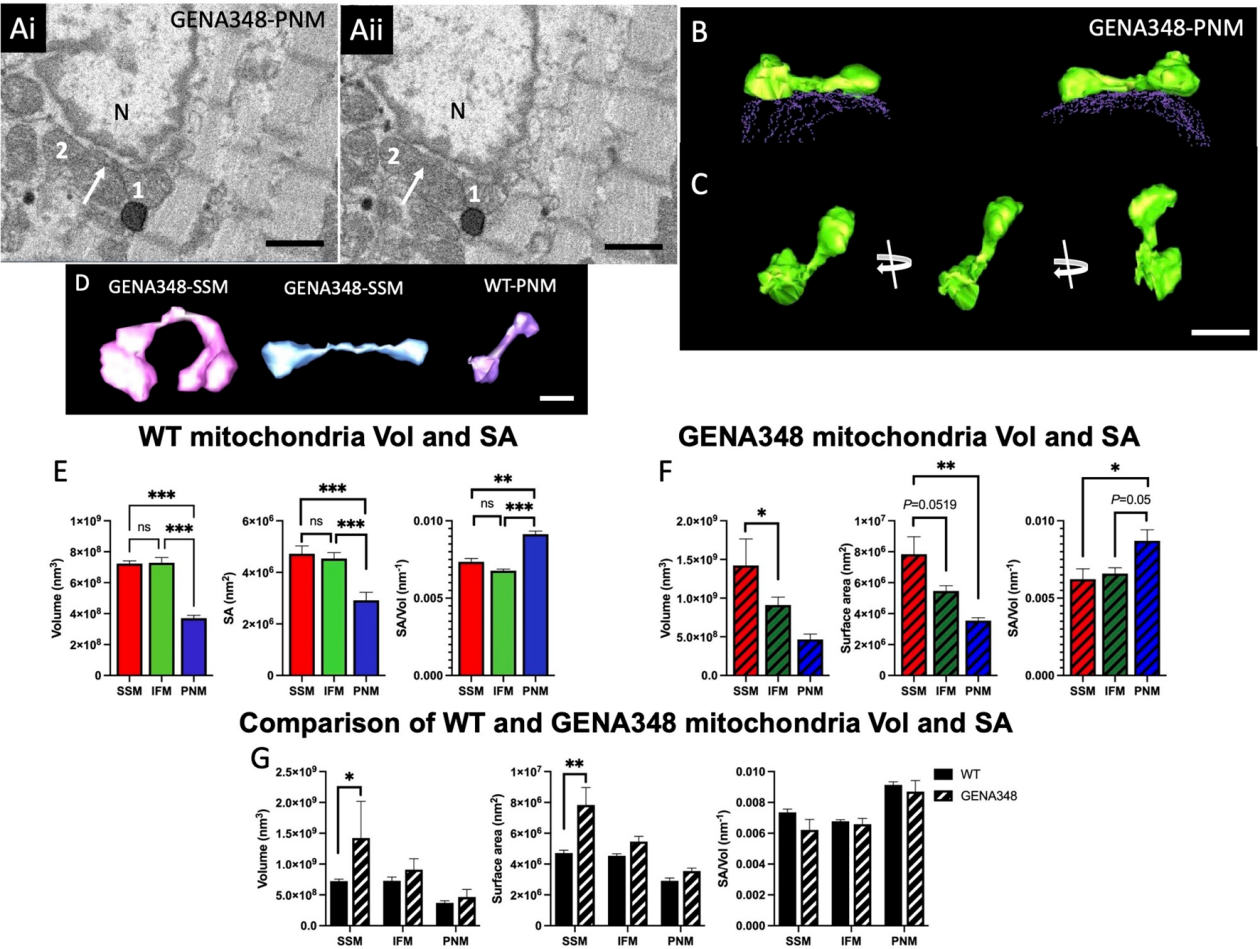
Morphometric analysis of mitochondrial subpopulations in the WT and GENA348 myocardium (apex). (**Ai**) Portion of a serial image showing two mitochondrion (labelled 1 and 2) adjacent to the nucleus, N. The arrow indicates a small projection extending from mitochondrion 1. (**Aii**) Shows the same area but several images (100 nm in Z-direction) further into the SBF-SEM stack showing an extension from mitochondrion 2 (white arrow). (**B**) Segmentation of the two mitochondria labelled 1 and 2 reveals that the projections connect forming a tunnel between the two mitochondria leading to a ‘dumbbell’ shape structure (green isosurface). The nuclear envelope is shown as dashed contours (purple). Portions of each mitochondria and ‘nanotunnel’ are in direct contact with the nucleus. (**C**) Rotation of the 3-D reconstruction of the nanotunnel showing that the tunnel is approximately 1 µm in length. Scale bar = 1 µm. (**D**) Examples of 3-D reconstruction nanotunnels identified in the WT and GENA348 mitochondrial populations. Scale bar = 1 µm. (**E**) Comparison of the WT Vol and SA for each subpopulation determined that PNM are smaller than SSM or IFM, with no difference between SSM and IFM. The SA/Vol ratio of the PNM is greater than SSM or IFM. Mean values (± s.d.) for the WT mitochondrial volumes are; 7.23 ± 0.31 × 10^8^ nm^3^ (SSM); 7.29 ± 0.59 × 10^8^ nm^3^ (IFM) and 3.70 ± 0.34 × 10^8^ nm^3^ (PNM); with a mean SA for each subtype of 4.72 ± 0.31 × 10^6^ nm^2^ (SSM); 4.54 ± 0.23 × 10^6^ nm^2^ (IFM); 2.92 ± 0.31 × 10^6^ nm^2^ (PNM). Data were analysed using a one-way ANOVA (with Tukey’s multiple comparison test) for each mitochondrial subtype from n = 3 animals. *P < 0.05, **P < 0.01, ***P < 0.001. (**F**) In the GENA348 cardiomyocytes SSM are larger than IFM and PNM (both Vol and SA). Analysis of SA/Vol shows a similar profile to WT mitochondrial subpopulations with PNM having the largest SA/Vol ratio. Mean values (± s.d.) for the GENA348 mitochondrial volumes are; 14.23 ± 3.44 × 10^8^ nm^3^ (SSM); 9.10 ± 1.02 × 10^8^ nm^3^ (IFM) and 4.66 ± 1.21 × 10^8^ nm^3^ (PNM); with a mean SA for each subtype of 7.84 ± 1.13 × 10^6^ nm^2^ (SSM); 5.46 ± 0.34 × 10^6^ nm^2^ (IFM); 3.55 ± 0.19 × 10^6^ nm^2^ (PNM). (**G**) A comparison of the morphometric parameters show GENA SSM are larger than WT. SA/Vol shows no difference between the WT and GENA348 subpopulations. Data analysed using a two-way ANOVA (Sidaks multiple comparison test). Data presented as mean ± SEM; **P < 0.01, ***P < 0.001, ****P < 0.0001.

In addition to the tubular projections we also identified ‘nanotunnels’, morphological adaptations suggested to be an alternative to direct fusion between adjacent mitochondria facilitating content mixing between non-adjacent mitochondria^21^; a nanotunnel is defined as tubular connection between two mitochondrion that are not necessarily adjacent to each other. We identified four nanotunnels in the WT PNM and GENA348 SSM and PNM populations (but not IFM); although in each instance, the ‘connected’ mitochondria were in close proximity to each other. Figure 3A illustrates how two GENA348 PNM mitochondrion (labelled 1 and 2), separated only by one mitochondrion, when viewed in a serial image each have a tubular projection. Segmentation of both of these mitochondria determined that the two projections join to form a nanotunnel (Fig. 3B). As shown in Fig. 3B,C portions of the nanotunnel are juxtaposed to the nuclear envelope, which may also indicate an adaptive development to improve diffusional properties for efficiency of metabolite exchange and substrate usage as well as inter-mitochondrial communication^22^. Examples of the other nanotunnels we identified are shown in Fig. 3D; the connecting tubule length ranged from 1.1 to 1.91 μm and with diameters of 0.28 to 0.33 μm. It is not possible to practically quantify the number of nanotunnels within each dataset without reconstructing all the mitochondria to determine whether projections ‘connect’ to form nanotunnels. Consequently, we cannot determine whether there is an increased frequency of nanotunnels in the disease model or rule out that there may be nanotunnels that span distances > 1.9 μm within cardiomyocytes. However, it is interesting to note that the mean width of the nanotunnels we identified are in the same range as the mitochondria with a projection, as reported in Table 2.

### SSM are enlarged in the GENA348 myocardium

We calculated the volume (Vol) and surface area (SA) of the segmented individual mitochondrion in the WT SBF-SEM datasets (n = 3, one‐way ANOVA), which revealed no inter-animal differences for the SSM, IFM or PNM morphological parameters; (raw data shown in Supplementary Fig. 1). A comparison of each mitochondrial subtype determined that PNM are smaller than both SSM and IFM, with no statistical difference between the SSM and IFM; Fig. 3E. However, a comparison of the SA/Vol (a parameter usually associated with diffusional properties) reveals that the SA/Vol of PNM is 1.24 × and 1.35 × greater compared to IFM and SSM respectively.

Analysis of the segmented GENA348 SSM, IFM and PNM showed inter-animal variation between the mitochondrial subtypes (raw data shown in Supplementary Fig. 2); however, a common pattern (which differs to WT morphology) between subtypes for each animal was identified for both Vol and SA, whereby SSM are larger than IFM which are larger than PNM (Supplemental Fig. 3). Combining the GENA348 data for n = 3 shows that the mean Vol and SA for SSM are larger than PNM (*P* = 0.0184), Fig. 3F; there is also a linear size trend whereby SSM > IFM > PNM. Similarly, the SSM SA is larger than PNM (*P* = 0.0047) and consistent with the individual GENA348 datasets. The mean data (n = 3) are representative with SSM trending larger than IFM, (*P* = 0.0519) and IFM larger than PNM (*P* = 0.0987). As shown for WT PNM the SA/Vol relationship of GENE348 PNM is larger compared to IFM or SSM.

A comparison of the WT and GENA348 subtype parameters revealed that the GENA SSM volume is 1.97 × and SA is 1.7 × larger than the WT (Fig. 3G). Compared to WT there is no difference between the GENA348 IFM and PNM Vol and SA or the SA/Vol ratio of the GENA348 mitochondrial subpopulations. Therefore, while the GENA348 IFMs have more projections the size of the tubules has not significantly altered the mean Vol compared to WT. Although, it was noted that the mean SA value of GENA348 IFM is 1.2 × larger than WT, a value that is close to significance (*P* = 0.061). Similarly, while there is no change to the PNM Vol parameters there is a trend for an increased SA in the GENA348 (*P* = 0.0749). In summary, we have determined that in both (i) WT and GENA348, PNM are smaller than IFM and SSM and (ii) PNM have a larger SA/Vol. However, a key difference is that GENA348 SSM are enlarged compared to WT SSM.

### Increased mitochondrial density and clustering in the GENA348 myocardium

**Figure 4:**
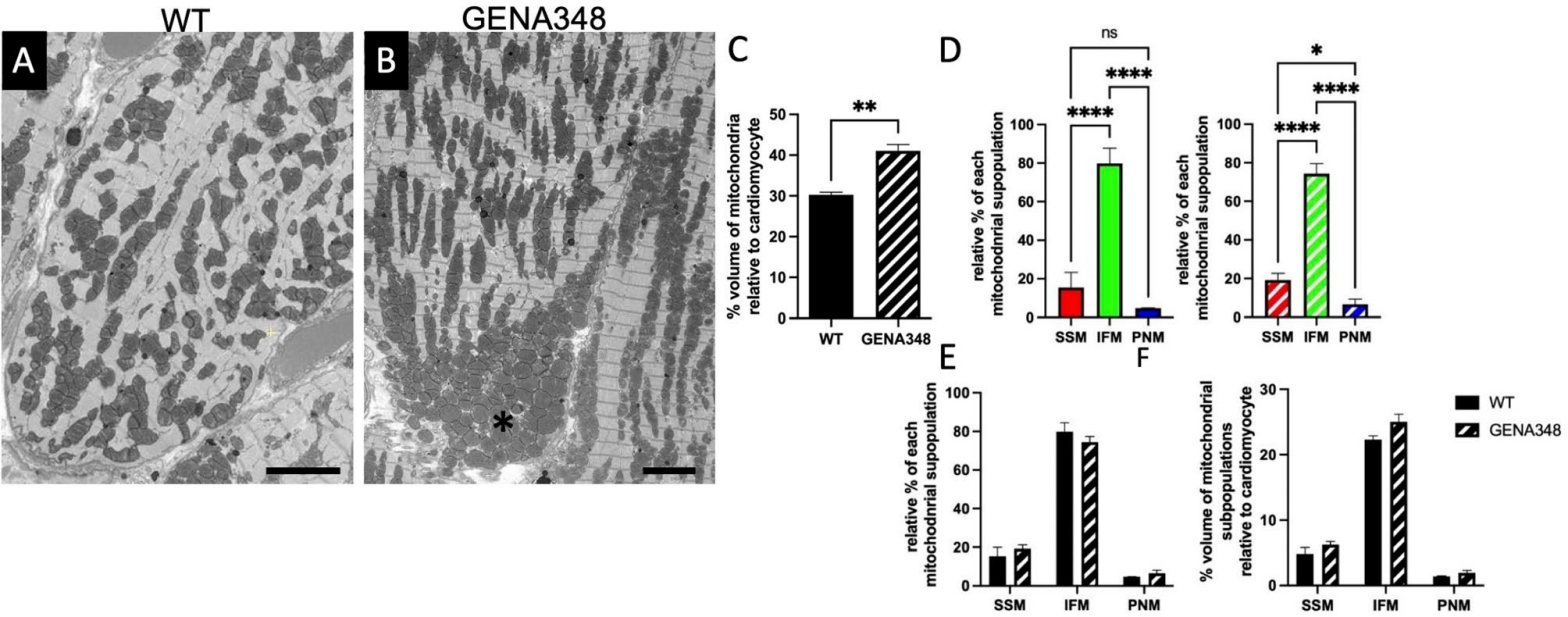
GENA348 myocardium has an increased mitochondrial density compared to the WT. (**A**,**B**) Portions of serial images from the WT SBF-SEM datasets (**A**) and GENA348 samples highlighting areas (*) of mitochondrial clustering. Scale bar = 5 μm. (**C**) Analysis of mitochondrial density using a stereological approach sampling SBF-SEM data sets (volume sampled per dataset = 58.4 × 58. 4 × 47.0 μm, n = 3 per group). Mitochondrial volume relative to the cardiomyocyte volume density for WT and GENA348 (mean ± SEM; two-tailed unpaired Student *t* test; **P < 0.01). (**D**) Analysis of the mitochondrial subpopulation densities relative to the total mitochondrial shows that IFM are the largest mitochondrial subpopulation in both the WT (left) and GENA348 (right) population. In the WT cardiomyocyte the density of SSM and PNM are similar but in the GENA348 myocardium there are more SSM than PNM. (**E**) There is no difference in the density of the WT and GENA348 mitochondrial subpopulations relative to the cardiomyocyte density. (**F**) All mean values of the GENA348 subpopulations are greater than WT, but do not reach significance (2-way ANOVA; Sidaks multiple comparison test) which may be due to the density increase being distributed across all groups.

During the segmentation process mitochondrial clustering in the GENA348 myocardium was observed (Fig. 4A,B) therefore, we next quantified mitochondrial density by applying a stereological method using macros developed by Mironov^23^ to the SBF-SEM datasets. WT mitochondria occupy 30.2 ± 1.1% of the cardiomyocyte volume compared to 41.1 ± 2.7% (*P* = 0.003) in the GENA348 myocardium (Fig. 4C). Applying the same approach we measured the density of the mitochondrial subpopulations in the WT and GENA348 datasets. The number of nuclei per volume analysed for WT and GENA348 was kept the same for comparative purposes. A limitation of investigating SSM, IFM and PNM separately is that the subpopulations of mitochondria are inter-connected^14^ and thus there is an element of subjectivity with respect to assignment of each subpopulation as they converge. Additionally, since only volumes containing a nucleus were analysed, the percentage of PNM are likely to be an overestimation and density of total number of mitochondria an underestimation. The majority of the mitochondria in the WT cardiomyocyte are IFM, 80.1 ± 7.2%, compared to SSM, 15.3 ± 7.2%, and PNM 4.7 ± 0.5%, with respect to the total mitochondria content (*P* < 0.0001). There is no difference between the SSM and PNM density (*P* = 0.0797) (Fig. 4D). IFM are also the major population in the GENA348 cardiomyocytes, 74.3 ± 5.3%, compared 19.2 ± 3.5% of SSM and 6.5 ± 2.8% PNM (*P* < 0.0001). A difference between the GENA348 and WT density subtype distribution is that the SSM population is greater than the PNM (*P* = 0.0186). A direct comparison of WT and GENA348 mitochondrial subpopulation densities show no significant change Fig. 4E. Further there is no change to the volume occupied by the subpopulations relative to the cardiomyocyte volume (Fig. 4F). The increase in density identified in Fig. 4C is likely distributed across the subtypes leading to the non-statistical increase, although the increase to the GENA348 IFM population is close to significance (*P* = 0.0552). In conclusion, another feature of the GENA348 model is (i) an increase to overall mitochondrial density and (ii) the relative density of GENA348 SSM to PNM is greater whereas in WT cardiomyocytes there is no difference between the subpopulations.

### There is an imbalance in mitochondrial fission–fusion axis in the GENA348 myocardium

**Figure 5:**
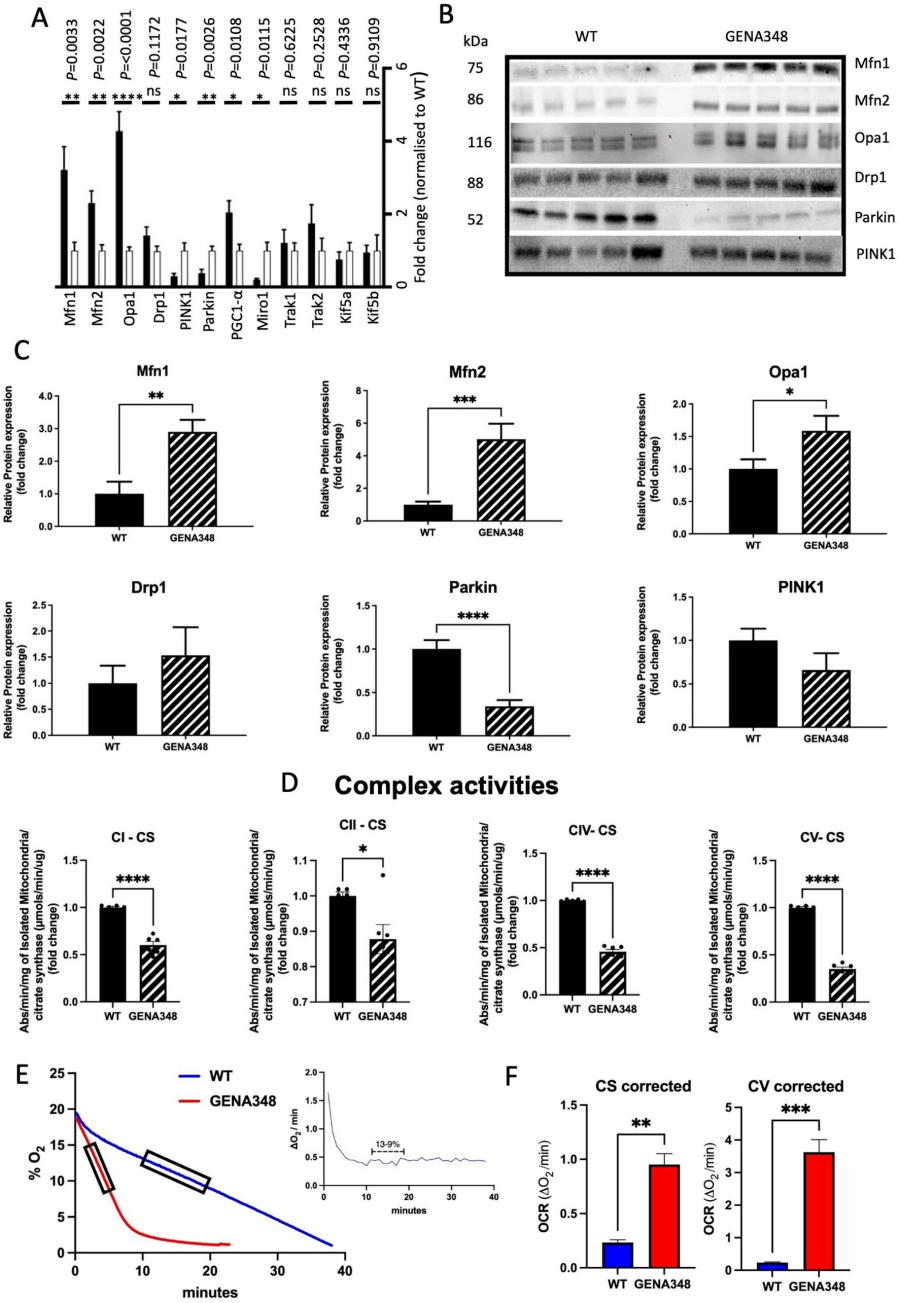
Molecular and functional remodeling of the GENA348 mitochondria compared to WT. (**A**) qPCR data illustrating fold changes to mRNA levels of proteins regulating mitochondrial dynamics, biogenesis, mitophagy and motility, white bars correspond to WT and black bars GENA348. (**B**) Western blots of key fusion and fission proteins and mitophagy markers PINK1 and Parkin (a method of total protein was employed for normalization rather than using a housekeeping protein^19,57^. (**C**) Western blotting (n = 5 per group) shows there is good agreement for the fission and fusion proteins and Parkin with the qPCR data. (**D**) Enzymatic assays indicate impaired activity of GENA348 Complexes (n = 5 per group). Data were corrected for citrate synthase activity CI; Complex I; CII, Complex II; CIV, Complex IV; CV, Complex V; (n = 5 per group). (**E**) Raw data indicating that the OCR is faster in the isolated GENA348 mitochondria. Rates were calculated in regions of steady state as shown in the inset and as previously described^29^ for each preparation. (**F**) Left; OCR data normalised to citrate synthase (CS) activity. Right; OCR data normalised to uncorrected Complex V (CV) activity relative to WT; both methods show that the OCR is faster in the GENA348 myocardium compared to WT. All data is presented as mean ± SEM; two-tailed unpaired Student *t* test; *P < 0.05, **P < 0.01, ***P < 0.001 and ****P < 0.0001.

To explore the mechanistic basis for the morphological changes to the GENA348 mitochondria we next investigated whether the expression levels of the proteins mediating mitochondrial size were altered in the GENA348 myocardium. As shown in Fig. 5A at the molecular level there is an increase in mRNA levels of the OMM fusion proteins Mfn1, Mfn2, and IMM Opa1, by 3.2-fold, 2.3-fold and 4.2-fold respectively, but no change to the fission protein Drp1; changes that would favour mitochondrial fusion and consistent with the formation of larger mitochondria. PINK1 and Parkin (components central for mitophagy, and regulating the turnover of Mfn1 and Mfn2) were both down-regulated 0.3-fold and 0.4-fold respectively, suggesting that the clearance of damaged mitochondria may be impaired. We also identified increased levels of PGC1-α (2.0-fold), a regulator of mitochondrial biogenesis and linked to induction of Mfn2 expression^24^. However, Miro1 (Rhot1) is down-regulated in the GENA348 mouse heart by 0.2-fold compared to WT; Miro1 mediates mitochondrial motility and forms part of the quality control process of mitophagy. Although there are no changes to transcript levels of TRAK1, KIF5A and KIF5B (proteins that form a complex with Miro1), the loss of Miro1 will impair mitochondrial movement as it is a crucial component for mitochondrial transport along microtubules, as demonstrated in a cardiomyoblast cell line^25^. While in mature cardiomyocytes it was thought that mitochondria are relatively static, there is evidence from multiple sources to indicate they are dynamic organelles that undergo mitophagy^26^. Therefore, the increase in mitochondrial content is consistent with increased levels of PGC1-α but also may be due to impaired mitophagy indicated by reduced levels of PINK1 and Parkin. Loss of Miro1 is also a potential contributory factor in the irregular distribution and turnover of mitochondria within the GENA348 cardiomyocytes. Western blotting of the main fission–fusion proteins agreed with the changes identified by qPCR (Fig. 5B,C). While levels of PINK1 levels were not significantly lower, the mitophagy marker Parkin is reduced at the protein level by 0.3-fold. Parkin recruitment by PINK1 is an essential step for ubiquitination of the OMM proteins on damaged mitochondria to instigate clearance by mitophagy^27^.

### Cardiac mitochondrial dysfunction is a feature of the GENA348 mouse

Investigations of mitochondrial function determined that the enzymatic activities of GENA348 Complex I, IV and V, underpinning OXPHOS and ATP production, are reduced compared to WT with an increase to Complex II activity. Specifically, Complex I is reduced by 0.86-fold (*P* = 0.0479); Complex IV reduced by 0.65-fold (*P* < 0.0001); Complex V reduced by 0.50-fold (*P* < 0.001) and Complex II increased by 1.26-fold (*P* = 0.0039). To normalise these data to mitochondrial content (particularly given the increase in density and PGC1-α) we corrected the activities to citrate synthase (CS) activity, a commonly used standard marker of mitochondrial content^28^. CS activity was increased by 1.42-fold (*P* = 0.0476) within the GENA348 mitochondria. After CS correction a reduction in Complex I, IV and V activity remains a feature of the GENA348 mitochondria but Complex II is now also decreased compared to WT (Fig. 5D). As shown in Fig. 5E we next measured the rate of mitochondrial oxygen consumption (OCR)^29^; a comparison of the gradients suggests that the GENA348 OCR is faster than WT. Normalisation to CS showed that the OCR in isolated GENA348 mitochondria is four-fold faster (*P* = 0.0023) compared to WT (Fig. 5F). In an alternative approach to normalising the OCR data we used the uncorrected enzymatic activity of Complex V activity which as shown in Fig. 5F, indicates that the GENA348 OCR rate is also significantly faster compared to WT (*P* = 0.0009). Here, we illustrate that the choice of normalisation marker does not alter the conclusion that the GENA348 mitochondria consume oxygen at a faster rate than WT.

### There are fewer mitochondria with regular cristae in the GENA348 myocardium

**Figure 6:**
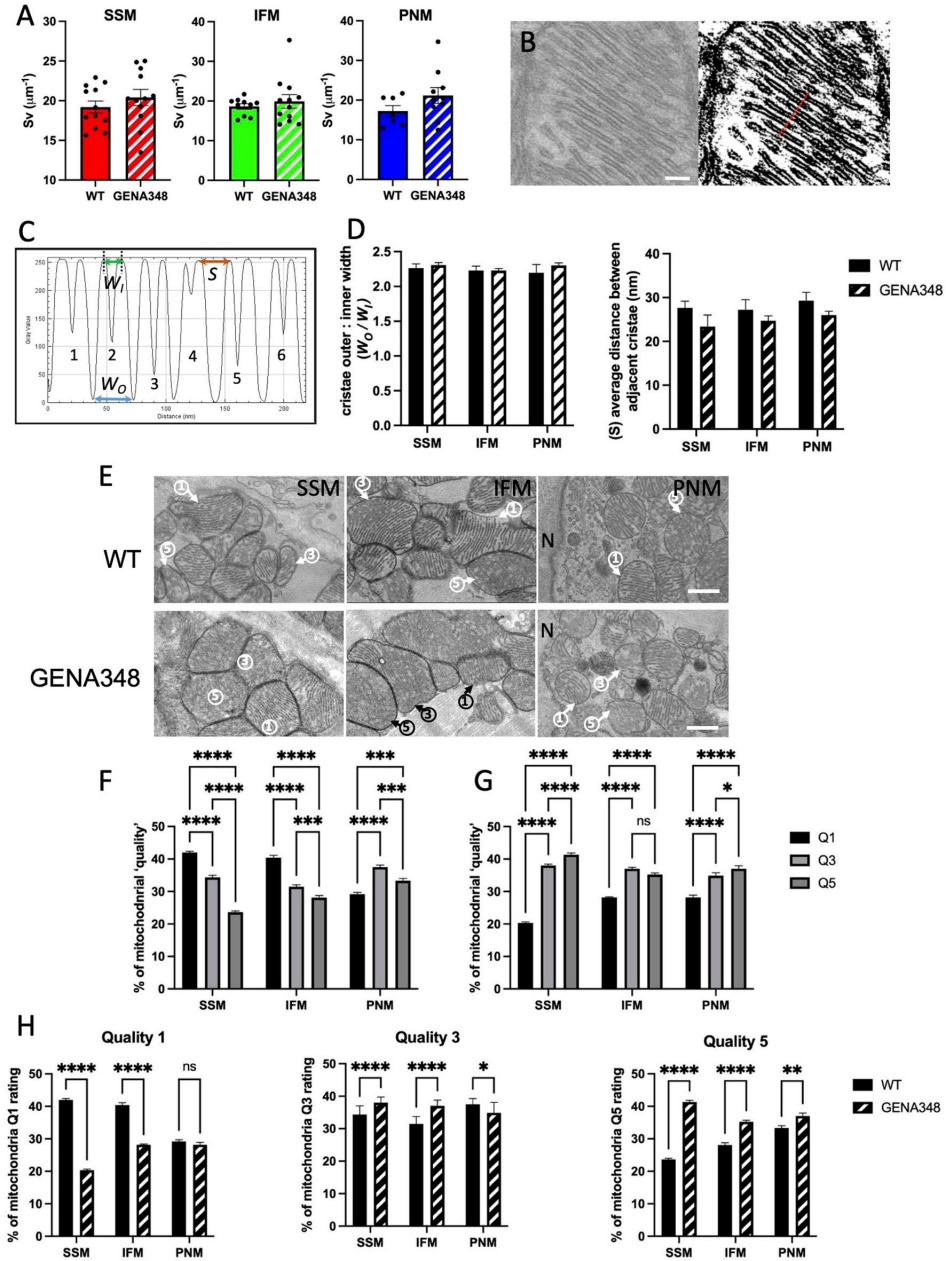
Comparison of cristae density and dimensions in each of the mitochondrial subpopulations using TEM images of thin sections of the WT and GENA348 myocardium. (**A**) There is no difference between the cristae density within the mitochondrial subpopulations. Each data point represents the mean of the cristae density for a mitochondrial subpopulation in each TEM image analysed. (**B**) Left: Example of a mitochondrion with ordered cristae (WT) used to analyse cristae dimensions. Right: The same mitochondrion after applying the (ImageJ) threshold function. Scale bar = 100 nm. The red line illustrates the position of the ‘line plot’ which spans 6 cristae. (**C**) Density analysis of the line spanning the cristae highlighted in (**B**) using ‘plot line’ function (ImageJ), each cristae is labelled 1–6. Each crista has a characteristic doublet representing the outer and inner membrane surface. Parameters measured were W_O_, the distance between the outer membrane leaflet; W_I_, the distance between the cristae inner membrane (lumen) and S, the distance between adjacent crista. (**D**) There is no difference between the WT and GENA348 cristae widths (W_O_/W_I_) or spacing (S); (see Table 2 for dimensions). (**E**) Examples of mitochondria in WT and GENA348 micrographs assigned according to ‘quality’; 1 = mitochondria has ordered cristae; 3 = mitochondria with areas of partial cristae organization; 5 = no visible cristae. *N* nucleus. (**F**) WT SSM and IFM mitochondrial subpopulations have a greater percentage of type 1 mitochondria with ordered cristae. (**G**) GENA348 SSM and PNM have more mitochondria with only partially ordered cristae and mitochondria without defined cristae. (**H**) A comparison of the ‘quality’ scoring between WT and GENA348 subpopulations shows that there are less ‘type 1’ mitochondria with more ‘type 3 and 5’ in the GENA348 SSM and IFM compared to WT. All data is presented as mean ± SEM. Data were analysed using a one-way ANOVA (with Tukey’s multiple comparison test) n = 3 animals per group.

Given the change to the Complex activities and OCR, we next investigated the cristae morphology using transmission electron microscopy. Applying stereological methods to the TEM images^23,30^, we determined that the cristae density for each mitochondrial subtype within WT and GENA348 mitochondria was unchanged (Fig. 6A). As shown in Fig. 6B,C we also measured the dimensions of the individual crista; the width between the crista outer membrane edges, W_O_, and the width between the inner membrane leaflet W_I_ (crista lumen) and spacing (S) between individual crista for SSM, IFM and PNM in each experimental group (see Table 2). There is no difference to the cristae dimensions or spacing within group subpopulations or when GENA348 cristae were compared with WT, Fig. 6D in keeping with the density analysis.

A caveat to both the cristae density and dimension analyses is that only mitochondria with clearly defined cristae, with no areas of cristae loss or disorganization were included. Therefore, a comparison has been made with potentially ‘healthy mitochondria’ within each experimental group. Therefore, we next classified the mitochondria in the TEM images according to cristae organisation as previously described e.g.^31^. We used an arbitrary scoring rating of 1, 3 and 5 where, 1 = ordered cristae; 3 = partially ordered cristae, and 5 = no discernible cristae respectively (Fig. 6E). WT SSM and IFM have the largest category of type 1 mitochondria whilst for WT-PNM type 3 is the largest group, Fig. 6F. In contrast, there are less of type 1, with more type 3 and type 5 mitochondria (i.e. more mitochondria with disordered cristae) in the GENA348 SSM and PNM subpopulations. GENA348 IFM have similar numbers of type 3 and 5 but also have less type 1 (Fig. 6G). A comparison of each ‘quality’ type between WT and GENA348 (Fig. 6H) shows that the number of mitochondria with ordered cristae is reduced in the SSM and IFM populations in the diseased model (*P* = < 0.0001), but with no change to the PNM population. Therefore, while there is no change to the cristae morphology of ‘type 1’ mitochondria, there are less of ‘type 1’ mitochondria with ordered cristae in both the SSM and IFM populations in the disease model, in keeping with the concept of impaired mitophagy and accumulation of dysfunctional mitochondria.

## Discussion

Here we report using a mouse model of diabetes presenting with hyperglycemia (but with no change to insulin levels), that both mitochondrial structural and functional remodelling occurs. Specifically, we have identified: (1) Morphological changes to the gross shape of the GENA348 mitochondrial subpopulations and (2) that there are more GENA348 mitochondria with tubular projections, with SSM having longer and wider tubules than in WT. (3) There is an increase in overall mitochondrial density and (4) increase to SSM size and relative mitochondrial density within the GENA348 cardiomyocytes. (5) GENA348 mitochondria have aberrant Complex activity and OCR compared to WT mitochondria. (6) The molecular data indicates that mechanistically these structural remodelling events and functional changes may be explained by a shift towards fusion, impaired mitophagy and motility further supported by (7) a reduction in the number of mitochondria with ordered cristae.

### GENA348 mitochondria exhibit multiple morphological differences compared to WT

Mitochondrial plasticity governing shape, size and distribution is intrinsically linked to function, with changes to morphology shown to reflect the energy demands of the cell, Ca^2+^ signaling and ROS production (recently reviewed by^32^), as well as in response to physiological stress and autophagy^33^. IFM pleomorphy in the heart is also associated with mechanically induced deformations and for facilitating electrical coupling through the mitochondrial network under physiological conditions^34^. Therefore, our shape analysis, while subjective, shows that in the WT myocardium there is a mix of oval and irregular shaped mitochondria in agreement with mitochondria as dynamic organelles with morphological adaptations linked to function^26^. An increase of irregular to oval GENA348 IFM may therefore represent a stress response and the development of early LV dysfunction with alterations to mechanical load^35^. Our novel results show that tubular projections are a feature of both WT and GENA348 mitochondria. However, the frequency of projections in the GENA348 SSM and IFM is greater in comparison to WT which coupled with the GENA348 SSM projections being longer than the WT SSMs may also suggest an increased need for communication between the mitochondria due to the diabetic stress. While it was not possible to quantify the nanotunnels in WT and GENA348 samples, it is noteworthy that Franzini–Armstrong and colleagues reported an association between nanotunnel frequency and stress describing an increased occurrence of nanotunnels in response to a calcium homeostasis imbalance due to a mutation in the ryanodine receptor (RyR2A4860G^+/−^)^36^. Nanotunnel formation is also linked to impaired fission–fusion with incomplete mitochondrial fission of mitochondrial tubules shown to generate anatomically similar structures to nanotunnels^37^. It is interesting that the width of the nanotunnels is similar to the tubular projections, and thus it is tempting to consider whether tubular projections particularly within the GENA348 SSM population may be pre-cursors to nanotunnel formation. A study by Wang et al. proposed a novel mechanism, (tested in NRK cells) whereby the formation of mitochondrial tubular projections, driven by KIF5B and fusion (Mfn1/2), underpins the formation of the mitochondrial network and communication^38^; Mfn1/2 were increased but not KIF5B (mRNA) in the GENA348 heart.

### GENA348 mice have enlarged SSM compared to WT

WT and GENA348 PNM while smaller than SSM and IFM, have a larger SA/Vol; a feature we suggest maybe optimised for diffusional properties between the PNM and nuclear envelope for both efficiency of substrate uptake and ATP production crucial for nuclear function/transcriptional activity^22^. As reviewed previously^4^ both mitochondrial swelling and fragmentation are reported in a range of cardiac pathologies. The inconsistencies to morphological changes between reports likely reflects the different methods of model generation, age, type and stage of the disease. Additionally, most previous reports have used 2-D EM images rather than 3-D for morphometric analyses where the size and shape will depend upon the orientation of the mitochondria and cut angle of the section. Notably, cardiac mitochondria from type II diabetic patients show a change in substrate usage and compromised OXPHOS in only SSM populations^39^. Therefore, it is significant that the GENA348 SSM Vol and SA are increased compared to WT and indicative that this remodelling is an early pathogenic event. The differential morphological effects upon the spatially distinct groups of mitochondria may be due to the close proximity between the SSM and sarcolemma and hence the more localized effects of external stimuli/stress compared to the other subpopulations, also reflected by the formation of longer and wider tubular projections.

### An imbalance in mitochondrial dynamics develops in the GENA348 myocardium

The increased levels of Mfn1/2 and Opa1 suggest a shift towards fusion. Mfn1 and Mfn2 as regulators of mitochondrial size have been demonstrated in numerous studies, and hearts deficient in Mfn2 exhibit cardiac hypertrophy and have small fragmented IFM^40^. Analysis of mRNA levels of Mfn2 in skeletal muscle of healthy, obese or diabetic patients suggests a link between depressed Mfn2 expression and insulin resistance^41^; results that are consistent with our data. Consequently, there is much interest in targeting mitochondrial fission–fusion as a therapeutic strategy for treating cardiomyopathies. However, our data showing increased levels of Mfn2 in the GENA348 myocardium concomitant with depressed Complex activities and altered OCR may indicate that increased fusion and larger SSM may not be beneficial in this context, or alternatively may represent an early adaptive remodelling event to combat pathophysiological events. Additionally, since Mfn2 is pleiotropic protein with functions beyond that of regulating mitochondrial shape^42^ changes in expression may impact on multiple signalling pathways.

### Increased mitochondrial density and clustering in the GENA348 myocardium

Our application of stereological methods to 3-D EM datasets showing that mitochondria occupy ~ 30% of the cardiomyocyte volume in WT mice, are in agreement with other studies using different approaches^43,44^. The absence of a statistical density change to the GENA348 SSM, IFM and PNM populations maybe due to the increase in density being distributed across all subtypes; though it is noteworthy that there are more SSM than PNM in the GENA348 myocardium. Interestingly, Glancy et al.^45^ identified a subset of SSM, paravascular mitochondria, that form clusters at the skeletal muscle sarcolemma adjacent to a capillary in order to enhance oxygen uptake, future work could therefore also consider evaluating remodelling of the GENA348 cardiac vasculature. Consistent with the GENA348 mitochondrial density increase is the up-regulation of PGC1-α, mediating biogenesis^46^, which may be a compensatory effect for the failing functional capacity of the existing mitochondria. However, the reduction of Parkin (at both mRNA and protein level), a regulator of mitophagy, also infers that there is an accumulation of damaged mitochondria, which would similarly contribute to an increase in mitochondrial density and explain the impairment of Complex activity. The depletion of Miro1, (a regulator of mitochondrial motility^25^), may also impact mitochondrial clearance processes as the mitochondria are unable to be transported for degradation; although the role of Miro1 in cardiomyocytes is not yet fully understood. In agreement, the ‘quality’ analysis showing an increase in mitochondria with either partially or no resolved cristae in the GENA348 cardiomyocytes compared to WT lends further support to the idea of mitochondrial ‘contagion’ due to the accumulation of dysfunctional mitochondria being associated with Parkin deficiency^27^. Bers and colleagues showed a link between mitochondrial movement and mitophagy, describing how mitophagy is centered around the perinuclear region of the cardiomyocytes and that transport of IFM towards the PNM location under conditions of stress is part of the mitochondrial quality control system^26^.

### GENA348 mitochondria have altered metabolism

Normalisation of mitochondrial functional data is common practice to correct for mitochondrial content, which may be altered under stress conditions with changes in biogenesis and/or to account for protein from non-mitochondrial sources. Here we employed CS to normalise the functional data, a commonly used approach^28^. Correcting the Complex activity in this way did not change the results of the raw data showing a depression of Complex I, IV and V activities; although Complex II activity is increased before correction and decreased afterwards. Complex II activity is linked to both ROS production and repression, depending upon the available substrate (alterations in substrate usage are a feature of various cardiac pathologies), other Complex activities and mitochondrial membrane potential^47^. In the heart differential effects upon Complex II activity have been reported during the various phases of ischemia–reperfusion injury and preconditioning^48^. A recent study has suggested CS may not always be accurate marker of mitochondrial content indicating that more detailed investigations are needed to investigate tissue specific markers for normalizing mitochondrial content using e.g. mitochondrial-targeted LC–MS/MS^49^. While conducting an MS analysis for normalization purposes is outside the scope of this current study we also tested correcting the OCR to Complex V activity; this method led to the same result showing that GENA348 mitochondria consume oxygen at a faster rate than WT. Correction against the other uncorrected Complex activities (including Complex II) also led to the same result (not shown).

We noted a similar change to OCR in hearts from a model of high fat feeding suggestive of a common pathology, although that model exhibited hyperglycaemia and hyperinsulinemia and increased Complex II activity, which was suggested to be associated with increased fat consumption leading to a shift to increased fatty acid β-oxidation^29^. A maladaptive hallmark of type II diabetes associated with insulin resistance is a shift in the balance towards fatty acid oxidation with increased fatty acid uptake^50^. However, there is a general assumption that heart failure in the absence of diabetes favours a shift towards increased glucose utilisation, although there are inconsistencies in the literature, as reviewed by Karwi et al.^51^. In the case of the GENA348 mouse model an explanation for the change in oxygen consumption rate may be the impairment of the glycolysis cycle due to the *Gck*-mutation leading to a shift towards fatty acid β-oxidation, and consequently aberrant O_2_ consumption rates, explaining the similarities to the OCR profile observed in the high fat feeding model^29^.

### There are less mitochondria with ordered cristae within the GENA348 myocardium

Cristae organization within mitochondria underpins OXPHOS and ATP production^52^. We report the cristae maximum diameter is ~ 36 nm and internal diameter (cristae lumen) of ~ 16 nm, data consistent with studies of human fibroblasts using cryo-electron tomography^53^ suggesting that the chemical fixation method employed here has not affected morphology. Comparing only ‘type 1’ mitochondria we identified no change to cristae dimensions. A study from Riva et al. reported that aging had no effect upon cardiac cristae morphology (IFM or SSM)^54^. Based upon the quality analysis our data may suggest that it is the loss of organized cristae coupled with impaired mitochondrial clearance leading to the accumulation of ‘type 3 and 5’ mitochondria that may be the main factor underlying mitochondrial dysfunction in the GENA348 myocardium, rather than discrete changes to cristae morphology.

### GENA348 mice develop mild LV dysfunction

Finally, while *Gck*-MODY2 patients are generally asymptomatic it is interesting to note that the GENA348 mice present with a reduction in the E:A ratio and increased IVRT (Table 1) parameters that are suggestive of diastolic dysfunction which is an early manifestation of type II diabetes^55^. Since there is no change to the ejection fraction (EF), the GENA348 mice may be developing early stage heart failure with preserved ejection fraction (HFpEF); a form of heart failure which now accounts ~ 50% of heart failure cases and is associated with comorbidities such as obesity and diabetes^56^. Therefore, while the GENA348 mouse may not completely recapitulate the human disease the data here may suggest that *Gck*-MODY2 patients warrant increased monitoring for developing cardiac complications.

In summary, this study has determined that cardiac mitochondrial remodelling of structure and function, is a feature of hyperglycaemia (without hyperinsulinemia) in a mild form of type II diabetes. Mechanistically, our data point towards a combination of adaptations to combat the diabetic stress such as mitochondrial biogenesis and morphological changes with more SSM and IFM tubular projections, and larger SSM; with stalled mitophagy leading to the accumulation of malfunctioning mitochondria with disorganised cristae and inefficient OCR. Given, the established link between mitochondrial dysfunction and heart failure development in patients, with and without diabetes, the data here provide new insights as to early adaptive/pathological remodelling events that occur in a model of mild diabetes.

## Methods

### GENA348 model and characterization of the cardiac phenotype

All animal work was performed in accordance with the United Kingdom Animals (Scientific Procedures) Act (1986) and relevant guidelines and regulations. The study was conducted with Institutional approval from The University of Manchester Animal Welfare and Ethical Review Board and in accordance with the ARRIVE guidelines. The GENA348 mice were generated and phenotyped as previously described^17^. At 6 months of age an Acuson Sequoia 256 cardiac ultrasound system with 15L8 transducer probe (14 MHz), in M-Mode, was used to measure morphological parameters as detailed in Table 1. Serum was collected and insulin levels measured (ALPCO Mouse Insulin ELISA Kit, 80-INSMS-E01, E10). Fasted glucose levels were analysed using an Accu-Check (Aviva) blood glucose meter. Animals were sacrificed by cervical dislocation and physiological parameters measured. The ventricles were either taken immediately for electron microscopy and oxygen consumption rates (OCR) or frozen in liquid nitrogen and stored at − 80 °C for further analysis.

### Biochemical analyses

Protein expression levels at the transcript level were assessed by RT-qPCR employing standard methods (n = 5 per group). All primers were purchased from Qiagen (Supplementary Table S1). qPCR was carried out using the Brilliant III Ultra-Fast SYBR green qRT-PCR master mix (Agilent Technologies) according to manufacturer’s instructions on a 7500 Fast Real-Time PCR System (Applied Biosystems). Relative expression of each gene was calculated relative to GAPDH using the ΔΔCt method. Protein level changes were analysed by Western blotting (n = 5 per group). We employed the stain free method, as we and others have previously described^19,57^, using a total protein loading method as an internal standard to normalize loading between gels instead of a housekeeping protein. Antibodies were purchased from Abcam or Santa Cruz (Mfn1, abcam ab57602; Mfn2 abcam ab56889; Opa1, SC-30572; Drp1, SC-32898; PINK1, SC-33796; Parkin, ab77924).

### Mitochondrial isolation and complex enzymatic activities

Mitochondria were isolated from 50 mg of fresh tissue using the Abcam kit (Ab110168) and either used immediately for measuring OCR as we have previously described^29^ or stored at − 80 °C. Complex I, II, IV and V enzymatic activities were measured using Abcam kits (Ab109908, Ab109721, Ab109909, Ab109714 respectively) and data collected using a Thermo Labsystem microplate reader. Citrate synthase activity was measured using the MitoCheck Citrate Synthase Activity Assay Kit (Cayman Chemical Company).

### Analysis of mitochondrial morphology

We employed SBF-SEM to generate 3-D morphological data. Tissue was taken from the apex of each heart (WT and GENA348, n = 3 per group) and fixed according to our published methods^19^, and data collected using FEI Quanta 250 FEG SEM/Gatan 3View system as described previously^58^. Serial images were collected for a voxel size of 13.5:13.5:50 nm (X:Y:Z). Approximately 90 mitochondria of each type (SSM, IFM and PNM), were segmented (IMOD software^59^), sampled from a minimum of 3 different cardiomyocytes (~ 800 mitochondria per dataset). To minimise bias we segmented ‘clusters’ of each mitochondrial subpopulation where possible.

### Analysis of mitochondrial density using unbiased stereological point counting

To calculate the mitochondrial density we used the method of unbiased stereological ‘point counting’ employing macros developed by Mironov^23^ with Fiji^60^ to estimate the volume of the serial images occupied by cardiomyocytes and the mitochondria within the cardiomyocytes. Portions of the serial images were sampled in the X–Y plane firstly from the upper left-hand side (through images 10–310), upper right-hand side (330–630 images) and lower middle portion (650–950) (areas labelled 1, 2 and 3; Supplemental Fig. 4). Two grids, red and green, were overlaid on the image stack; the area between the points in the red and green lattice being 13.368 × 10^6^ nm^2^ and 2.674 × 10^6^ nm^2^ respectively, sampling mitochondria points in both grids every 20 images (every 1 µm). Points in the green grid falling on mitochondria were counted. Similarly, points in the red grid falling on any part of a cardiomyocyte were counted; but points falling outside the boundaries of a cardiomyocytes e.g. extracellular matrix, fibroblasts, blood vessels were not counted. To prevent edge effects objects touching left and bottom borders of white boxes were excluded from counting. For each portion (i.e. 1, 2 or 3), the number of points counted for each grid was tallied and the following equation used to calculate the percentage of the cardiomyocyte volume occupied by mitochondria:

$$\frac{{{\text{total}}\;{\text{ no}}{.}\;{\text{ of }}\;{\text{points}}\; ({\text{mitochondria}})\; \times \; {\text{green}}\;{\text{ grid}}\;{\text{ area}} \; \times \;({\text{no}}{. }\;{\text{of }}\;{\text{slices}}\; \times \;50\;{\text{nm}})}}{{{\text{total}}\;{\text{no}}{.}\;{\text{of }}\;{\text{points}} \;({\text{cardiomyocyte}}) \; \times \; {\text{red}}\;{\text{ grid}}\;{\text{area}}\; \times \;({\text{no}}{. }\;{\text{of }}\;{\text{slices}} \; \times \; 50\; {\text{nm}})}}\; \times \;100.$$

Therefore, for each dataset three density values were calculated for areas 1–3 and the mean ± SD was then calculated for each dataset. The overall mean for each experimental group was then calculated and an unpaired Student’s *t* test (two-tailed) was used to compare the mitochondrial density in the WT myocardium compared to the GENA348.

The same stereological approach was employed to analyse the SSM, IFM and PNM densities separately, using a different counter number for each subtype. While the same volume and grid sizes used were for the total mitochondrial density analysis, equivalent non-overlapping volumes (1, 2 and 3) were selected through the datasets to contain a nucleus in order to be able sample the PNM. A one-way ANOVA (Tukey’s multiple comparison test) was used to determine variation in subpopulation density for each animal (n = 3) and between WT and GENA348.

### Analysis of cristae density using unbiased stereological point counting

Ultrathin sections of 70 nm were cut using Reichert Ultracut microtome in an arbitrary orientation for analysis by TEM (Thermo Fisher Talos L120C) prepared from the blocks used for 3View as described above. Images were recorded at 120 kV acceleration voltage by Ceta camera at 13,500 × for each biological sample (WT and GENA348, n = 3 per group) for areas containing SSM, IFM and PNM as defined in Fig. 1A,B. Surface density of cristae per mitochondria volume were estimated using linear and point test probes according to Saltykov^30^. The method of intersection and point counting was applied to the TEM images in ImageJ using the multipurpose grid macro^23^ with an appropriate grid tile density. Points falling within mitochondria and lines intersecting with cristae were counted (Supplemental Fig. 5) for each micrograph. The total counts for mitochondria and cristae intersections for each mitochondrial subpopulation per biological sample were tallied. The formula to calculate the surface volume (${\hat{\text{S}}}v$) occupied by the cristae was then applied (formula taken from^61^):

$${\hat{\text{S}}}v(Y,ref) = \frac{{2 \cdot \sum\nolimits_{i = 1}^{n} {Ii} }}{{l{/}p \cdot \sum\nolimits_{i = 1}^{n} {Pi} }}$$

*Ii*; number of intersections with cristae; *Pi*; number of points falling within a mitochondria; *l*/*p* is the value calculated from the grid parameters corresponding to the ‘test line per any point’ generated upon selection of the tile density. Mitochondria that did not contain clearly defined cristae were excluded (Fig. 6E, type 3 and 5). The total number of mitochondria analysed were 66 SSM, 70 IFM and 35 PNM (WT) and 66 SSM, 63 IFM and 60 PNM (GENA348).

### Individual cristae dimensions

Using the same TEM images as for the cristae density analysis the ImageJ ‘Threshold’ function was applied as described previously^18^ so that cristae membranes appeared black (Fig. 6B). The ‘Plot Profile’ function was used to generate a grey scale profile of the cristae morphology (Fig. 6C). Using the measurement tool in ImageJ the dimensions of each cristae and spacing between adjacent cristae were recorded for each micrograph and mitochondrial subtype. The parameters W_O_, W_I_ and S were tallied for each mitochondrial subpopulation for each animal and a mean value calculated (n = 3). A one-way ANOVA (Tukey’s multiple comparison) was employed to investigate whether the cristae parameters varied between subpopulations with each group i.e., WT and GENA348 and then between experimental models.

### Statistical analyses

All data are reported as the mean ± (SEM). Unpaired two-tailed Student *t* test, one-way ANOVA or two-way ANOVA were applied as indicated. Outcomes were considered as significant when *P* < 0.05. GraphPad Prism 9 was used for all statistical analyses.

## Supplementary Information

Supplementary Information 1.

Supplementary Information 2.
