# Supplementary material for: Differential remodelling of mitochondrial subpopulations and mitochondrial dysfunction are a feature of early stage diabetes
Source: Sci Rep. 2022 Jan 19;12:978. doi: 10.1038/s41598-022-04929-1 (PMC8770458; doi:10.1038/s41598-022-04929-1)

Differential remodelling of mitochondrial subpopulations and mitochondrial dysfunction are a feature of early stage diabetes

Rajab et al

SUPPLEMENTAL INFORMATION

Western blots:

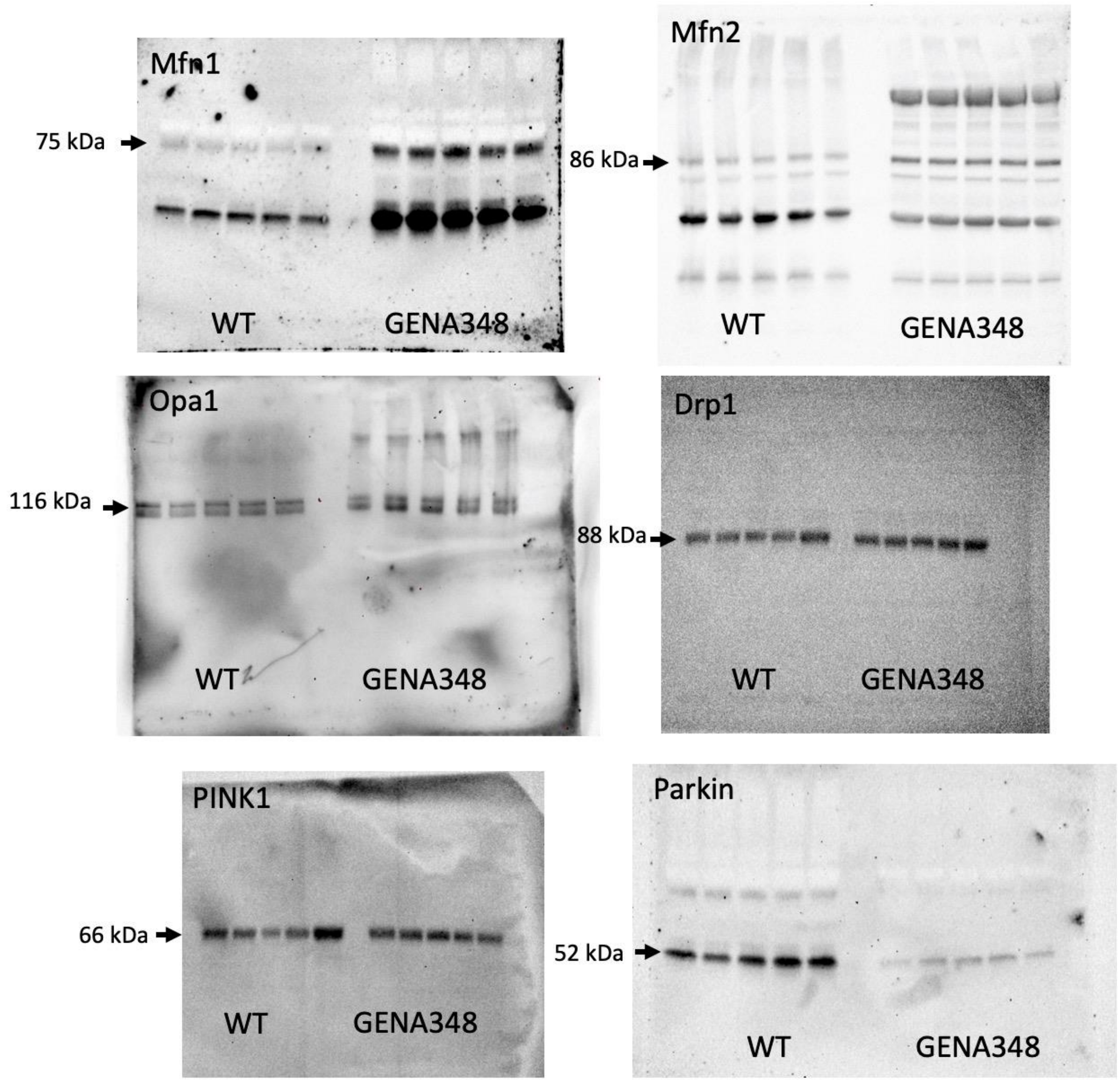

Supplement: Supplementary file 1 — Supplementary Information 1. [file 41598_2022_4929_MOESM1_ESM.pdf]
