# Supplementary material for: Differential remodelling of mitochondrial subpopulations and mitochondrial dysfunction are a feature of early stage diabetes
Source: Sci Rep. 2022 Jan 19;12:978. doi: 10.1038/s41598-022-04929-1 (PMC8770458; doi:10.1038/s41598-022-04929-1)
Supplement: Supplementary file 2 — Supplementary Information 2. [file 41598_2022_4929_MOESM2_ESM.pdf]

Differential remodelling of mitochondrial subpopulations and mitochondrial dysfunction are a feature of early stage diabetes

Rajab et al

SUPPLEMENTAL FIGURES

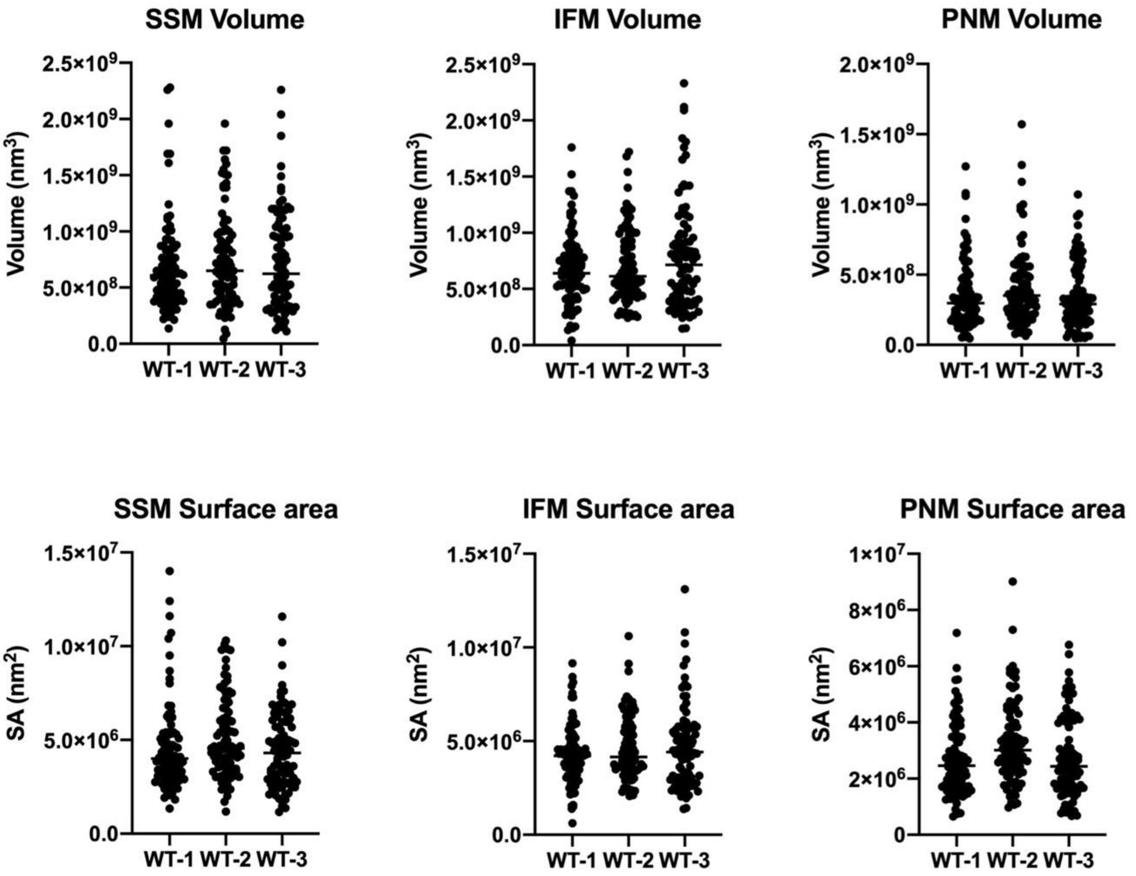

Figure S1: Individual data points of the cardiac mitochondrial morphological parameters of WT mice (6 months old). Approximately, 270 mitochondria were individually segmented per group per dataset and the volume and surface area calculated using the IMOD function. There is no inter-animal variation between WT mitochondrial subtype (SSM, IFM, PNM) for volume (nm^3) and surface area (nm^2).

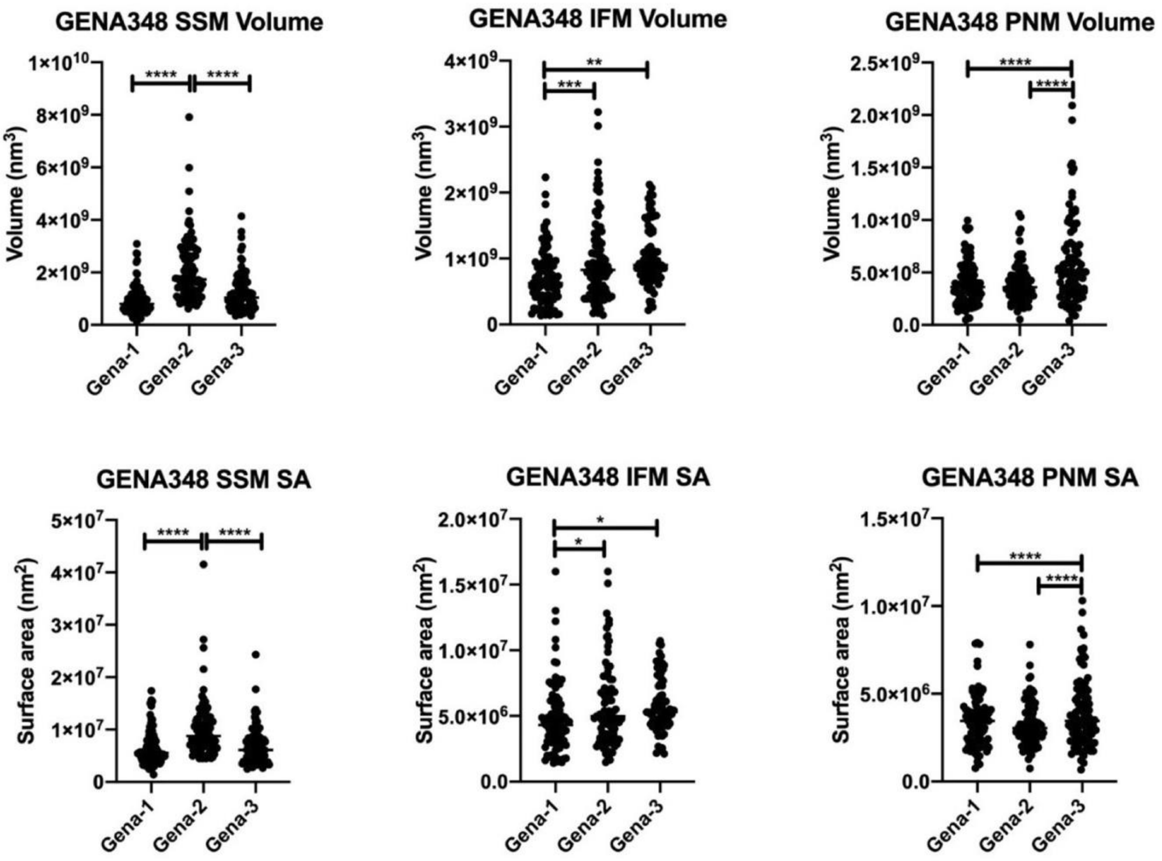

Figure S2: Individual data points of the cardiac mitochondrial morphological parameters of WT mice (6 months old). GENA348 mice exhibit inter-animal variation between the morphological parameters. (One-way ANOVA; *$P \leq 0.05$ and **$P \leq 0.01$, ***$P \leq 0.001$ and ****$P \leq 0.0001$).

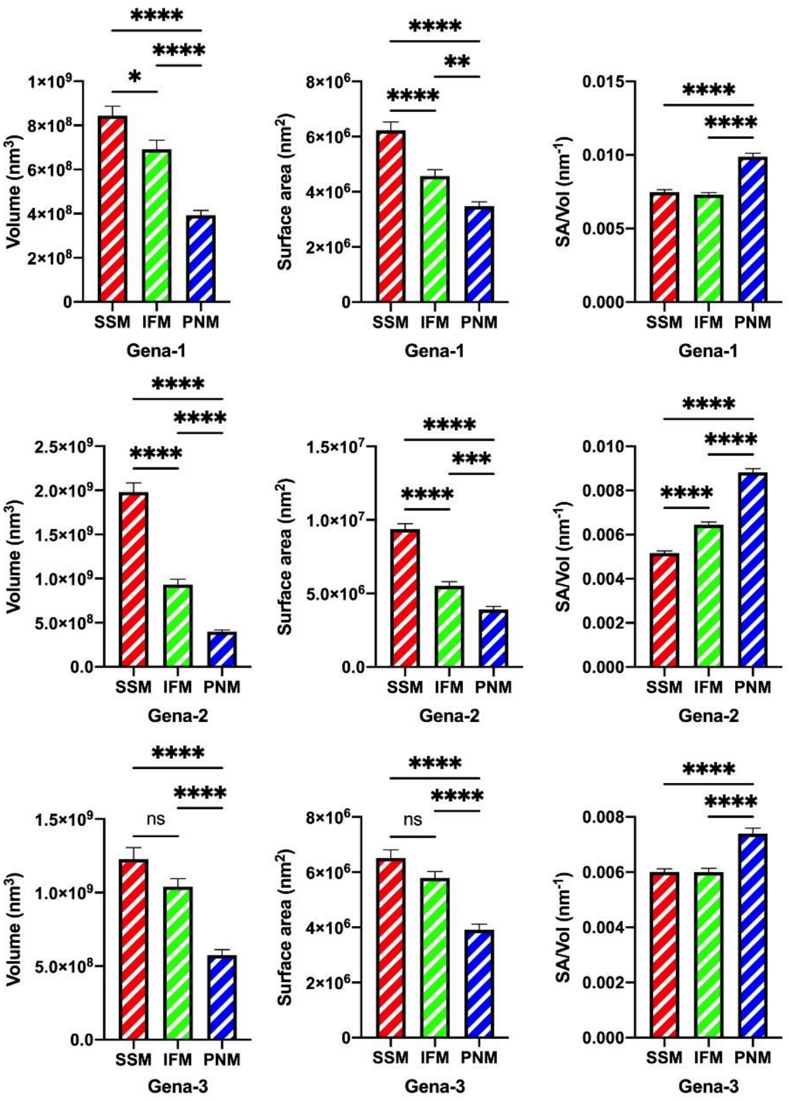

Figure S3: Analyses of the individual datasets for each GENA348 animal (Gena-1, Gena-2, Gena-3) identifies that the SSM are largest size compared to the other mitochondrial subpopulations. There is a common size distribution of SSM>IFM>PNM for all animals. *Note* for Gena-3 there was not a significant difference between the SSM and IFM but the IFM volume is trending smaller $P = 0.0797$; for the SA $P = 0.1140$. As with the WT mitochondria the SA/Vol parameter is largest for the PNM. (One-way ANOVA, Tukey's multiple comparison test; data is presented as mean ± SEM, *$P \leq 0.05$ and **$P \leq 0.01$, ***$P \leq 0.001$ and ****$P \leq 0.0001$).

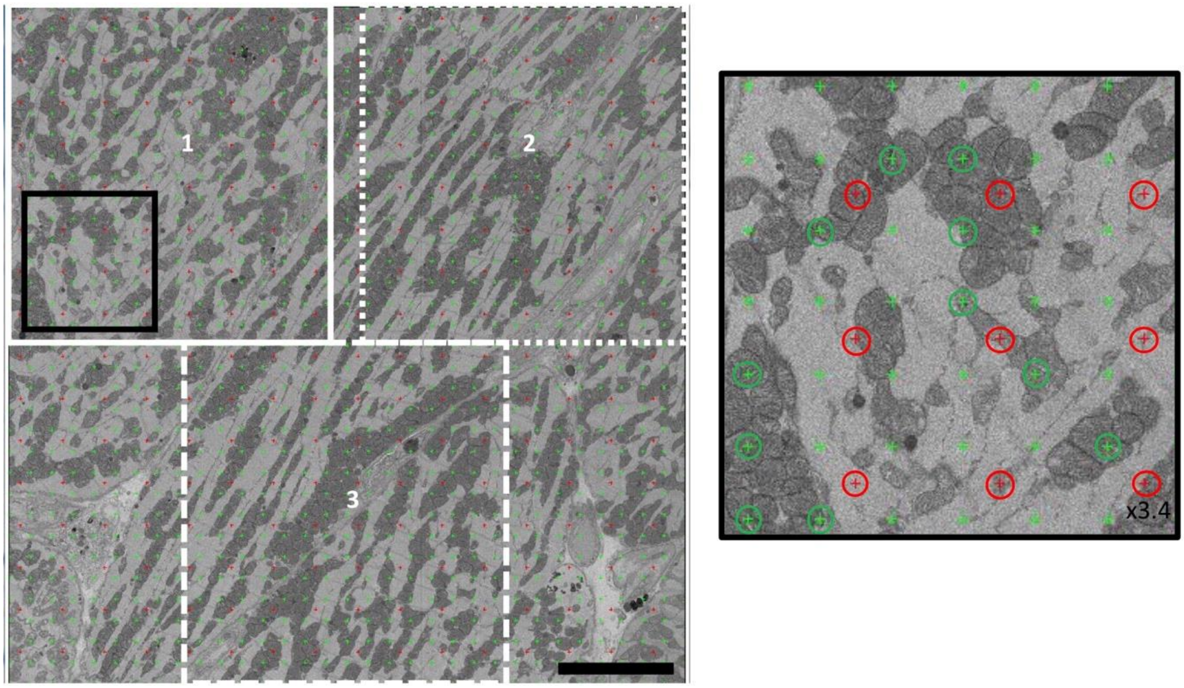

Figure S4: Mitochondrial density analysis

The three areas in (*left image*) sampled through the stack are indicated by the dashed white boxes numbered 1, 2 and 3. The green and red crosses are the two overlaid grid matrices. Image J generates area information between points on each grid: red 13.368 x 10^6 nm^2 and green: 2.674 x 10^6 nm^2. Scale bar = 10 µm. The area in the black box (*right image*) has been magnified 3.4 times to show details of the grids. Red points falling anywhere within a cardiomyocyte are counted for calculating the volume (cut-depth in the z-direction is 50 nm) occupied by cardiomyocytes within the tissue, whereas only those points in the green matrix that fall on mitochondria are counted to calculate the volume occupied by mitochondria.

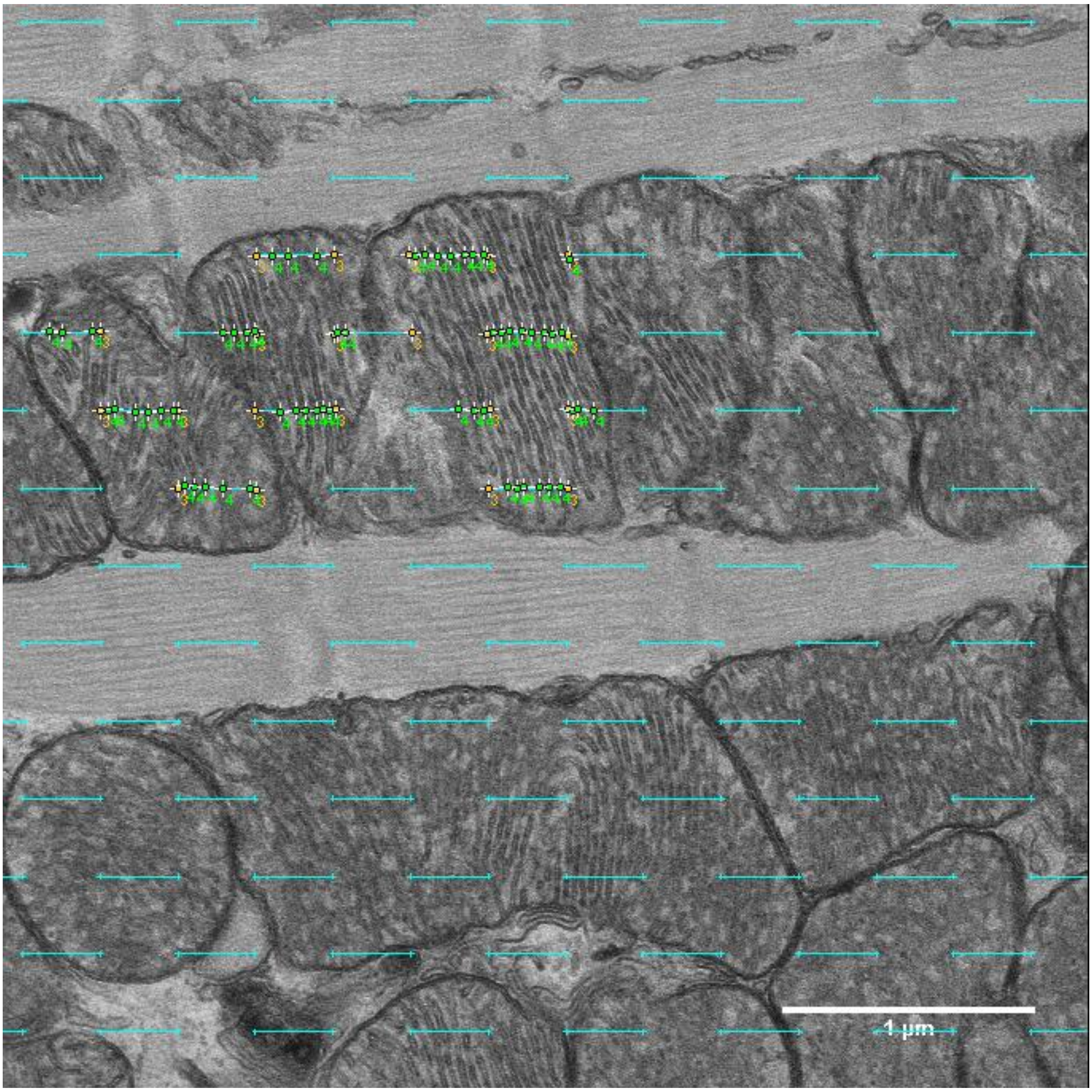

Figure S5 Cristae density analysis
TEM image of an area of IFM (GENA348), with the grid tile for stereological point counting overlaid (cyan). The end-points of the grid lines falling on a mitochondrion were counted indicated by the brown counter point (labelled 3). The green counter points (labelled 4) indicate where the line intersects with a crista.

Table S1

RT-qPCR: Primers were purchased from Qiagen

Target gene	Primer	Catalogue number
Opa1	Mm_Opa1_1_SG	QT00162085
Drp1	Mm_Dnm1L_2_SG	QT01166809
Mfn1	Mm_Mfn1_1_SG	QT00167839
Mfn2	Mm_Mfn2_1_SG	QT00134295
Gapdh	Mm_Gapdh_3_SG	QT01658692
PGC1- α	Mm_Ppargc1a_1_SG	QT00156303
PINK1	Mm_Pink1_1_SG	QT00111349
Parkin	Mm_LOC641034_1_SG	QT01278823
RHOT1	Mm_Rhot1_1_SG	QT00153706
TRAK1	Mm_TRAK1_va.1_SG	QT01562078
TRAK2	Mm_TRAK2_1_SG	QT01758918
KIF5A	Mm_Kif5a_1_SG	QT00252511
KIF5B	Mm_kif5b_1_SG	QT00142275
